# Ectopic Lymphoid Follicles in Multiple Sclerosis: Centers for Disease Control?

**DOI:** 10.3389/fneur.2020.607766

**Published:** 2020-12-08

**Authors:** Austin Negron, Olaf Stüve, Thomas G. Forsthuber

**Affiliations:** ^1^Department of Biology, University of Texas at San Antonio, San Antonio, TX, United States; ^2^Department of Neurology, University of Texas Southwestern Medical Center, Dallas, TX, United States; ^3^Neurology Section, Veterans Affairs North Texas Health Care System, Medical Service, Dallas, TX, United States

**Keywords:** germinal center, GCR, ectopic lymphoid follicles, ELF, follicular T helper cells, TFH, B cell, Th17 (T helper 17 cell)

## Abstract

While the contribution of autoreactive CD4^+^ T cells to the pathogenesis of Multiple Sclerosis (MS) is widely accepted, the advent of B cell-depleting monoclonal antibody (mAb) therapies has shed new light on the complex cellular mechanisms underlying MS pathogenesis. Evidence supports the involvement of B cells in both antibody-dependent and -independent capacities. T cell-dependent B cell responses originate and take shape in germinal centers (GCs), specialized microenvironments that regulate B cell activation and subsequent differentiation into antibody-secreting cells (ASCs) or memory B cells, a process for which CD4^+^ T cells, namely follicular T helper (T_FH_) cells, are indispensable. ASCs carry out their effector function primarily via secreted Ig but also through the secretion of both pro- and anti-inflammatory cytokines. Memory B cells, in addition to being capable of rapidly differentiating into ASCs, can function as potent antigen-presenting cells (APCs) to cognate memory CD4^+^ T cells. Aberrant B cell responses are prevented, at least in part, by follicular regulatory T (T_FR_) cells, which are key suppressors of GC-derived autoreactive B cell responses through the expression of inhibitory receptors and cytokines, such as CTLA4 and IL-10, respectively. Therefore, GCs represent a critical site of peripheral B cell tolerance, and their dysregulation has been implicated in the pathogenesis of several autoimmune diseases. In MS patients, the presence of GC-like leptomeningeal ectopic lymphoid follicles (eLFs) has prompted their investigation as potential sources of pathogenic B and T cell responses. This hypothesis is supported by elevated levels of CXCL13 and circulating T_FH_ cells in the cerebrospinal fluid (CSF) of MS patients, both of which are required to initiate and maintain GC reactions. Additionally, eLFs in post-mortem MS patient samples are notably devoid of T_FR_ cells. The ability of GCs to generate and perpetuate, but also regulate autoreactive B and T cell responses driving MS pathology makes them an attractive target for therapeutic intervention. In this review, we will summarize the evidence from both humans and animal models supporting B cells as drivers of MS, the role of GC-like eLFs in the pathogenesis of MS, and mechanisms controlling GC-derived autoreactive B cell responses in MS.

## Introduction

Multiple sclerosis (MS) is a neuroinflammatory autoimmune disease affecting nearly 2.3 million people globally (1). MS most commonly presents as episodes of neurological dysfunction followed by periods of clinical recovery known as remission. The accumulating damage resulting from the persistent repetition of relapse and remission is thought to eventually lead to a continuous phase of increased neurological dysfunction and disability without remission, known as secondary-progressive MS (SPMS). About 10% of patients immediately enter this phase after clinical onset in form of primary-progressive MS (PPMS) (2).

Evidence from human samples as well as from the animal model of MS, experimental autoimmune encephalomyelitis (EAE), has established that multiple cell types contribute to disease pathogenesis, with CD4^+^ T cells as the primary drivers of autoimmune pathology. However, the remarkable clinical success of Rituximab (RTX), a B cell-depleting monoclonal antibody (mAb) targeting CD20, challenged this long-held assumption, demonstrating that the role of B cells in MS may have been underappreciated (3). This proposition is further supported by studies showing that B cells are a major target of previously established disease-modifying therapies (DMTs), and specifically, that positive therapeutic responses are strongly associated with the elimination of pathogenic B cell subsets. The advent and efficacy of B cell-depleting therapies (BCDTs) has necessitated the reevaluation of the mechanisms underlying the pathogenesis and progression of MS.

Despite the considerable success of B cell-targeting therapeutics, clinical outcomes remain varied, similar to previously established DMTs (4). More importantly, the progressive forms of MS are refractory to nearly all currently approved DMTs. Most likely, the inability to halt disease progression is in large part a consequence of our incomplete understanding of the mechanisms responsible for progressive MS.

Along these lines, highly organized structures resembling secondary lymphoid organs (SLOs), known as ectopic lymphoid follicles (eLFs), were initially described in the 1980s and subsequently reported as a common feature of several chronic inflammatory autoimmune diseases (5–8). It is thought that these structures facilitate the perpetuation of autoreactive B cell responses. Interestingly, meningeal eLFs are found in a substantial proportion of SPMS patients, and aggregates of B and T cells were also observed in PPMS and RRMS patients, however, these notably lack features of more developed follicles such as follicular dendritic cells (FDCs), distinct T and B cell zones, and high endothelial venules (HEVs) (9–12).

In this review, we will summarize the progress made in understanding mechanisms of MS immunopathology, with particular emphasis on the role of eLFs as drivers of disease progression, cell types potentially involved in eLF development in MS. Furthermore, we will discuss treatments either currently available or in development that specifically target molecular or cellular mediators of eLF formation or function. Lastly, we will discuss key questions that remain unanswered.

## The Germinal Center Reaction

SLOs, such as the spleen and draining lymph nodes (DLNs), are specialized structures within which T cell- and B cell-dependent immune responses initiate and develop/mature. This is due to their ability to support germinal center (GC) reactions. GC reactions primarily serve to refine the B cell component of the adaptive immune response through selection and expansion of high-affinity B cell clones and subsequent differentiation into either ASCs, such as plasmablasts (PBs), and plasma cells (PCs), or into memory B cells (13–17). ASCs are effectors that function in both primary and subsequent immune responses. PBs are typically short-lived and serve to neutralize an acute threat by infectious pathogens, while PCs are long-lived, and reside in sites that are specially equipped to support their persistence (18). Memory B cells are rapidly activated upon secondary antigen encounter (19).

GCs are compartmentalized into a dark zone, within which B cell clones proliferate and undergo affinity maturation, and a light zone, where B cells undergo selection, differentiation, or are directed to return to the dark zone to undergo further rounds of affinity maturation and proliferation (16).

CD4^+^ T cells, specifically follicular T helper (T_FH_) cells, are principal orchestrators of this process and direct B cell fate decisions through the provision of surface-bound and soluble stimulatory and inhibitory signals (20–22). Additionally, several of these signals, such as interleukin-21 (IL-21) and CD40L, influence class-switch recombination (CSR), thus directing the nature of the B cell effector response. FDCs are a second specialized cell type that display antigen bound in form of immune complexes or with complement and therefore provide B cell receptor (BCR)-mediated survival signals to high affinity B cell clones.

Following resolution of the primary response, circulating and resident memory T and B cells are on stand-by for secondary antigen encounters, upon which they can undergo rapid differentiation and restoration of effector function. Importantly, these encounters can also result in the development of new GCs.

## MS Pathogenesis

### CD4^+^ T Cells in MS

MS has long been thought to be primarily mediated by autoreactive CD4^+^ T cells directed against central nervous system (CNS) antigens, such as myelin basic protein (MBP), myelin oligodendrocyte glycoprotein (MOG), or aquaporin-4 (AQP4) (23, 24). The pathogenic role of T cells is undisputed and is based mostly on the following observations: (1) The EAE model of MS can be induced by adoptively transferring myelin-reactive T cells into a healthy recipient animal; (2) the association of MS with human leukocyte antigen (HLA) DRB1^*^15:01; (3) the exacerbation of MS following treatment with an altered peptide ligand of myelin basic protein (MBP) that activated MBP-reactive T cells and led to disease exacerbations; (4) the *de novo* onset and the re-activation of MS during immune checkpoint inhibitors for cancer therapy; (5) the beneficial effects of T cell depleting pharmacotherapies, such as alemtuzumab, or therapies that sequester T cells out of the CNS, such as natalizumab; (6) the clonal expansion of CD4^+^ T cells infiltrating the CNS (25–35).

The importance of CD4^+^ T cells has been substantiated by studies from both humans and the animal model of MS, EAE. Indeed, CD4^+^ T cells are enriched in lesions of MS patients and EAE studies further revealed two pathogenic T helper subsets important for disease: interferon gamma (IFN-γ)-producing type 1 T helper (T_H_1) cells and IL-17 producing type 17 T helper (T_H_17) cells (36). In line with this assertion, both IFN-γ and IL-17 are detected in the lesions of MS patients (37). IFN-γ also positively correlates with increased disease activity and increased disability (38). Moreover, T_H_1 cells were found localized in CNS lesions in MS patients and are also increased in the CSF of RRMS patients during relapse compared with remission (39).

Taken together, experimental evidence from human MS patients and experimental animal studies have led to a proposed mechanism in which an unknown trigger results in the aberrant activation of autoreactive CD4^+^ T cells in the immune periphery, after which these encephalitogenic CD4^+^ T cells enter the CNS from the choroid plexus (CP), are reactivated by local APCs in the CNS, and initiate a proinflammatory cascade that results in increased permeability of the blood-brain barrier (BBB), subsequent recruitment of proinflammatory immune cells, and subpial cortical damage (40).

### A Trail of Breadcrumbs: Initial Evidence of Antibody-Mediated B Cell Involvement

A potential role for B cells in the pathogenesis of MS was initially suggested by the discovery of IgM and IgG antibodies in the CSF of around 40% and 95% of MS patients, respectively (24, 41). Intrathecal IgM and IgG, which are collectively referred to as oligoclonal bands (OCBs), are considered a diagnostic hallmark of MS due to their association with disease activity and persistence throughout the entire course of disease. A study comparing the CSF immunoglobulin (Ig) proteome and the Ig transcriptome of B cells within the CNS showed a strong overlap, demonstrating that ASCs generated from clonally expanded B cells within the CSF are the major source of intrathecal OCBs (42–44). Consequently, B cells were thought to contribute to MS primarily via the production of autoreactive antibodies targeting CNS antigens. In support of this, IgM antibodies targeting myelin lipids have been identified in MS patients and the presence of these antibodies is associated with a more aggressive disease course (45). Moreover, there was evidence of substantial IgG and complement deposition, as well as the presence of macrophages containing myelin-bound antibodies in patients exhibiting the most common demyelination pattern, pattern II, which is present in 60% of MS patients (46, 47).

Surprisingly, in stark contrast to classically antibody-mediated autoimmune diseases such as myasthenia gravis or Goodpasture's syndrome, identification of a disease-specific antigenic target remains elusive, and accumulating evidence supports reactivity toward a variety of self-antigens, from ubiquitously expressed intracellular proteins to neurofilament proteins (24, 48–50). However, antibodies targeting viruses have also been observed, such as the MRZ pattern, which consists of antibodies targeting the measles, rubella, and zoster viruses (51). Moreover, evidence suggests that these reactivities may be unique for different patients (52). The contribution of autoantibodies was further challenged by the finding that plasmapheresis was primarily beneficial in patients exhibiting pattern II demyelination (53).

### BCDTs: Ushering in a New Age

A phase 2 clinical trial testing the efficacy of the B cell-depleting mAb RTX as a treatment for RRMS showed that RTX was able to suppress inflammatory disease activity and reduce relapse rates (54). The striking results vindicated the previously overlooked pathogenic relevance of B cells and in doing so challenged our understanding of the mechanisms involved in MS pathogenesis and ushered in a new wave of therapeutics specifically targeting B cells. Following RTX, three subsequent anti-CD20 mAbs, each slightly varying in structure and specificity, have been developed in an effort to optimize safety and therapeutic efficacy: ocrelizumab (OCR), ofatumumab (OFT), and ublituximab (UTX). Both OCR and OFT have been approved and UTX is currently undergoing phase 3 trials (ClinicalTrials.gov number, NCT03277248) (55).

However, the benefit of BCDTs went beyond their obvious clinical efficacy. By studying the compositional changes in the CSF and periphery associated with successful clinical outcomes to BCDTs, our understanding of the dynamic involvement of B cells in MS has greatly advanced. One of the most impactful observations contributing to this advancement was that the positive clinical responses elicited by BCDTs took place without alterations in intrathecal OCBs. While this could have been anticipated due to the lack of CD20 expression on mature PCs, it indicated that B cells primarily exert their pathogenic function not by autoantibody secretion, but rather by antibody-independent mechanisms such as antigen presentation and pro-inflammatory cytokine secretion (56).

Memory B cells can function as potent APCs and therefore may contribute to the reactivation of CNS-reactive CD4^+^ T cells due to their superior ability to capture and present antigens present at very low concentrations compared with dendritic cells (24, 57–63). In strong support of this, memory B cells are not only increased in MS patients but also display elevated surface levels of MHCII and the costimulatory molecules CD80 and CD86 (64–66). Furthermore, these cells secrete proinflammatory cytokines such as IL-6, GM-CSF, and TNFα upon restimulation (67–71). Although B cells and even some subsets of ASCs such as IgA^+^ PCs are capable of secreting anti-inflammatory cytokines such as IL-10, TGF-β, and IL-35, MS patients are abnormally deficient in these regulatory-type B cell subsets, which further amplifies the effects of the aforementioned proinflammatory cytokines (68, 72–77). Subsequent studies investigating the cell populations predominantly affected by BCDTs as well as previously existing DMTs also point to memory B cells as a major pathogenic B cell subset in MS (56).

Importantly, the discovery of bidirectional exchange of B cell clones between the CNS compartment and the periphery gave strong credence to the possibility that in MS patients, memory B cells contribute to MS pathology by acting as the APCs that reactivate encephalitogenic CD4^+^ T cells and subsequently produce proinflammatory cytokines that further contribute to inflammation and damage within the CNS (43, 78).

The remarkable success of BCDTs in treating MS is blunted however by heterogeneous clinical outcomes, and more-so by the inability of these treatments to halt advancement of disease progression (4). Even treatment with OCR and OFT, which have been approved for the treatment of PPMS and active SPMS, only slow rather than halt progression. However, the inability of these antibodies to cross the BBB may provide a possible clue to their failure in arresting disease progression (56).

### OCBs: Pathognomonic Yet Poorly Understood

Among the changes in our conceptual understanding of MS pathogenesis, it is now acknowledged that MS involves both peripheral as well as compartmentalized inflammatory processes in the CNS. While our understanding of the mechanisms leading to and sustaining compartmentalized inflammation remains largely incomplete this process is thought to be driven by tissue resident populations (12, 79–81).

OCBs are thought to be produced by ASCs derived from the local antigen-driven reactivation of memory B cells within the CNS, indicated by mutations highly concentrated within the CDR3 regions (82). This finding has been corroborated by other studies (83, 84). This evidence of a CSF-restricted humoral response demonstrates that B cells participate in and potentially contribute to compartmentalized inflammation seen during later disease stages (85).

Importantly, the discovery of B cell-rich follicles in the meninges of up to 40% of SPMS patients pointed to the possibility that these structures might be involved in the reactivation of encephalitogenic CD4^+^ T cells (12). Although initially not considered as a pathognomonic feature of MS, these aggregates correlate strongly with cortical pathology and disease severity in PPMS and SPMS patients. Moreover, the continual antigen-driven expansion of B cells in MS patients strongly implicated eLFs as a prospective driver of MS progression and warrants their investigation. Therefore, given their resemblance to eLFs seen in other chronic inflammatory conditions, these structures might offer a potential explanation as to the source of continual OCB production seen in MS.

## Ectopic Lymphoid Follicles in Multiple Sclerosis: Centers For Disease Control

The development of structures analogous to SLOs has been reported in peripheral tissues at sites of chronic inflammation, serving as a reservoir for autoreactive B and T cell reactivation. These structures are known by a variety of monikers, such as tertiary lymphoid organs (TLOs), ectopic lymphoid structures (ELS), and tertiary lymphoid tissues (TLTs), but will be referred to as eLFs in this review (86, 87).

eLFs support the continuous antigen-driven expansion of B cells in sites of chronic inflammation and are therefore a common feature of several B cell-mediated autoimmune diseases such as rheumatoid arthritis (RA), systemic lupus erythematosus (SLE), and Sjögren's syndrome (24). eLFs have been demonstrated in the meninges of approximately 40% of SPMS patients (10, 80, 88). Moreover, recent evidence suggested that these aggregates are not restricted to late disease stages but rather are also present in early stages of MS (12, 89). Indeed, meningeal inflammation strongly correlates with subpial cortical injury in nearly all disease stages.

While eLFs share structural and functional similarities with SLOs, the mechanisms underlying their initiation and establishment as well as the cellular players involved and required are quite different. Moreover, due to the specialized nature of the CNS, meningeal eLFs warrant special considerations that set them apart from eLFs in other disease settings. Molecular and cellular traffic to and from the CNS is stringently regulated by the blood-CSF and BBB, barriers that inadvertently provide a significant level of protection for eLFs established within this restricted tissue (90).

Here, we will detail (i) the similarities and differences regarding the establishment and maintenance of SLOs and eLFs, (ii) the unique nature of the CNS as a site of chronic inflammation and eLF formation, and (iii) current evidence supporting the potential role for eLFs in driving MS disease progression.

### SLO vs. eLF Establishment

SLOs are ideally suited entities for facilitating immune surveillance and the adaptive immune responses, namely the GC reaction. As a result of their importance in mediating such a nuanced and vital process, the development and location of these tissues is genetically preprogrammed. Broadly, SLO formation involves three main phases: the establishment of chemotactic gradients to facilitate B cell and T cell homing and clustering, stimulation of tissue remodeling and angiogenesis, and the formation of a stromal reticular network.

Lymphoid organogenesis is catalyzed by the interaction of lymphoid tissue-inducer (LTi) cells with lymphoid tissue-organizer (LTo) cells via the binding of lymphotoxin (LT)α_1_β_2_ to the LTβ receptor (89). This stimulates LTo cells to produce the chemokines CCL19, CCL21, CXCL13, and CXCL12, as well as growth factors such as VEGF-C and FGF2. The resulting chemotactic gradient facilitates immune cell homing and compartmentalization, while the growth factors stimulate the development of lymphatic vessels and HEVs, allowing B and T cell ingress. Importantly, the chemokines secreted by LTo cells also continue to recruit LTi cells, forming a positive feedback loop important for the maintenance of this process. LTo cells also begin to express intercellular adhesion molecule (ICAM)-1 and vascular cell adhesion molecule (VCAM)-1 in order to aid in immune cell retention upon entry. Finally, LTo cells will differentiate into FDCs, fibroblastic reticular cells (FRCs), and marginal reticular cells, which comprise the stromal reticular network (89, 91, 92).

The formation of eLFs follows the same basic developmental steps as the formation of SLOs, however the first key distinction is that eLF formation is triggered in response to inflammation and thus can occur in a variety of non-lymphoid tissues and the resulting structures are not encapsulated (90). In this context, immune cells have the capacity to function in a manner analogous to LTi cells. For example, in the context of pulmonary inflammation, the development of inducible bronchus-associated lymphoid tissue (iBALT) was dependent on T_H_17-derived IL-17 (90). B cells have also demonstrated this LTi-like ability in a model of colitis, but in a LTα_1_β_2_-dependent manner (93). In a similar fashion, the role of LTo cells is taken on by stromal organizer cells such as fibroblasts and endothelial cells that are activated by the inflammatory milieu. In addition to providing the aforementioned homeostatic chemokines, these activated stromal cells can also produce survival factors such as B cell-activating factor (BAFF) and cytokines capable of influencing the T cell response, such as IL-6 which promotes T_H_17 responses (93).

Partially due to their formation being initiated by inflammation, eLFs can form and dissipate quickly. As a direct consequence of this transient nature, eLFs can display significant organizational and cellular heterogeneity ranging from small and disorganized aggregates of B and T cells to highly organized structures containing compartmentalized T and B cell zones, HEVs, FDCs, and a developed stromal reticular network (10, 11, 80, 90, 91). It is important to note, however, that once the reticular network has formed and an eLF has reached an advanced state of maturation, eLFs become fairly stable and are less likely to dissipate (94). The dependency of eLFs on the inflammatory context is apparent in conditions such as RA, where inflammation in articular joints is chronic and promotes self-sustaining eLFs. Additionally, in diseases such as RA and myasthenia gravis, the presence of disease-specific autoantigens enables the long-term persistence of eLFs. Thus, the extent of organization of an eLF is a consequence of the extent and persistence of inflammation (87, 94, 95). Furthermore, in MS mature meningeal eLFs are exclusively found in SPMS patients as compared with PPMS and RRMS patients (10, 11, 80).

Importantly, smaller and less developed eLFs are still able to support typical GC-related B cell processes such as affinity maturation, proliferation, and differentiation (90). This might be a result of the inflammatory microenvironment, as well as of the tendency of eLFs to be comprised primarily of memory B and T cell populations, which differ from their naïve counterparts in regard to signaling requirements. Moreover, GC-related processes in eLFs can occur independently of T_FH_ cells, and are instead facilitated by a T_FH_-like population known as peripheral T helper cells, which lack the canonical T_FH_ cell markers CXCR5 and BCL6 (96).

Nevertheless, it must be noted that inflammation is not the sole prerequisite for eLF formation. Rather, the permissiveness of a tissue to the influx and aggregation of lymphocytes is an equally important consideration during this process (97, 98). This quality is particularly apparent in the context of MS, as the CNS is unique in its structural and circulatory properties, both of which can dramatically change in the context of inflammation.

### Immune Cell Access to the CNS: Keys to the Kingdom

The CNS is a vital system and the regulation of cellular and molecular influx and efflux is accordingly more complex than in most other tissues, a characteristic reflected in the structures within and the barriers surrounding it, e.g., the BBB. The CNS parenchyma is enveloped by the meninges, a structure consisting of the dura mater, the arachnoid mater, and the pia mater. The dura contains fenestrated blood vessels as well as lymphatic vessels, both of which facilitate trafficking of lymphocytes between the CNS and the deep cervical lymph nodes (dCLN). The two innermost layers, the arachnoid mater and the pia mater, are collectively known as the leptomeninges and are separated by the subarachnoid space, a cavity filled with CSF (99).

Produced by the CP, CSF plays an important role in remote immune surveillance of the CNS due to its role in the glymphatic system in which the interstitial fluid, which contains molecules drained from the parenchyma, is taken up by the CSF and flows via the lymphatic vessels into the dCLN. This is thought to be important for tolerance, as it facilitates the presentation of parenchymal self-antigens in the absence of inflammation (100). Additionally, it also provides a medium by which lymphocytes circulate within and surveil the subarachnoid space.

Lymphocyte entry to the CNS is regulated by two specific barriers: the BBB and the blood-cerebrospinal fluid barrier (BCSFB). The BBB, which separates the leptomeningeal and deep parenchymal capillaries from the perivascular subarachnoid and Virchow-Robin (VR) spaces, is made up of endothelial cells connected by tight junctions. In the parenchymal capillaries, cell infiltration of the parenchyma is further restricted collectively by the pia mater, the glia limitans, which is a thin barrier comprised of astrocytic endfeet, and the parenchymal basal lamina.

The BCSFB regulates entry to the CSF-filled ventricles from the capillaries embedded within the CP stroma. In contrast to the BBB, this barrier is comprised of the fenestrated endothelium of the choroidal capillaries, and the ependymal cells, which are connected by tight junctions.

The BBB and BCSFB restricts lymphocyte access through the dynamic expression of specific adhesion molecules such as VCAM-1 and ICAM-1. In steady-state conditions, these two barriers allow minimal lymphocyte entry. In response to inflammatory signals, these barriers can become more permeable, increasing infiltration by lymphocytes (89). Furthermore, the leakiness of these barriers increases efflux of molecules such as chemokines and cytokines, resulting in further recruitment of potentially proinflammatory immune cells (101). Collectively, these barriers stringently regulate entry and egress of cells as well as macromolecules such as antibodies.

### Evidence of eLFs in MS

In 1979, Prineas (9) observed what they described as “reticular-like cells embedded within lymphoid-like structures and lymphatic capillaries within old plaques” in the CNS of MS patients. Subsequently it was shown that lymphocytic aggregates, found in the meninges of SPMS patients, appeared proximal to subpial lesions and correlated with disease severity and progression (10, 80, 88). Following the seminal findings by Prineas, Magliozzi, and Serafini, Lucchinetti et al. showed that perivascular T and B cell infiltrates could also be detected in acute and RRMS patients proximal to cortical plaques; however limited tissue availability prevented probing for other cell types characteristic of eLFs, such as FDCs (12, 89).

Interestingly, eLFs found in MS patients resemble those described in other chronic inflammatory autoimmune diseases such as RA, SLE, and Sjögren's syndrome. Moreover, the CSF of MS patients during disease relapses contains elevated levels of LTα and CXCL13, both of which are critical for lymphoid organogenesis, and the latter of which also correlates with the levels of intrathecal Ig and the frequency of B cells and PBs in the CSF. Furthermore, these follicles have also been reported to contain CXCL13, FRCs, and FDC-like CD35^+^ cells as well as HEVs (10, 90, 102).

The presence of B cell clusters surrounded by T cells makes meningeal eLFs ideal environments to facilitate GC reactions. Indeed, high-throughput Ig repertoire analyses of B cell clones from paired CNS and SLOs showed that antigen-driven affinity maturation can occur within the CNS (103). This view is further supported by the expression of activation-induced cytidine deaminase (AID), a required transcription factor for affinity maturation in the GC, in B cells from the CSF (104). Proliferating Ki67^+^ centroblasts have also been observed in the CSF but not the peripheral blood of MS patients, further indicating a compartmentalized GC reaction (105). Additionally, Ig repertoire analyses show a higher degree of somatic hypermutation, specifically in the CDR3 region, in CSF-derived IgM and IgG compared with those from peripheral blood, indicating antigen-driven affinity maturation within the CNS of MS patients (78, 82). The cytokine milieu in eLFs specifically supports these processes and the survival of B cells. The high concentration of BAFF, a potent B cell survival factor, is particularly notable, due to its ability to rescue self-reactive B cells from deletion (106). In RA, the abundance of survival factors such as BAFF has been attributed to the resistance of eLFs to BCDTs such as RTX (107). It is important to note that meningeal inflammation observed in early stage MS has been associated with pronounced subpial cortical pathology and is associated with more aggressive disease course (80). In light of these findings, and further supported by the correlation between meningeal inflammation and cortical pathology throughout all stages of MS, it is plausible that meningeal eLFs could serve as a supportive niche for the reactivation and persistence of autoreactive CD4^+^ T cells and memory B cells, thereby representing an insidious mechanism driving disease progression (24).

### Catch Me If You Can: Hurdles in Studying eLFs in MS

Despite the evidence detailed above, the ability to concretely demonstrate the relationship between eLFs and progression is mired by three critical limitations.

In MS, most observations are derived from analyzing post-mortem tissue samples, which are understandably limited in their availability. Importantly, these samples are typically obtained at later stages of disease when inflammation is possibly less pronounced (89). Therefore, while heterogeneous observations between patients regarding cellular composition and structural organization can partly be explained by the transient nature of eLFs, it is more likely a consequence of differences in disease stage and varying degrees of residual CNS inflammation between patients (91).

Conceivably, EAE studies may provide a viable alternative model to study eLFs. Indeed, EAE models have provided critical insights into the immunological mechanisms involved in MS and all therapeutics (such as natalizumab) have been developed as a direct result of EAE studies (108). An additional advantage is that the disease manifestations, including the involvement of specific cell types, can be adjusted based on the immunogen as well as the strain of mice. But despite their proven merit, current EAE models remain incomplete models of MS (90).

In regard to studying eLFs in the CNS, only few EAE models are able to form eLFs similar to those observed in humans (90). Even so, these models exhibit substantial variability, both between models and within the same model. The kinetics of eLF formation and maturation is a major factor in this, since the relatively short disease courses used may not provide enough time for eLF maturation.

Another limitation involves inter-species differences. One of the cell types strongly associated with eLF formation and subsequent GC-like responses is the T_FH_ subset. In humans, T_FH_ cells are substantial producers of CXCL13, a cytokine that facilitates eLF formation and maintenance as well as recruitment of CXCR5-expressing B cells. Murine T_FH_ cells, however, do not produce CXCL13 and instead parenchymal and stromal cells are the primary producers of this cytokine (109, 110). While interspecies differences like these are not uncommon by any means, a recent study might impart physiological relevance to this discrepancy: using a model of EAE in which MOG-specific T_H_17 cells are adoptively transferred to naïve mice, a model known to yield a high frequency of eLFs correlating to disease severity, Quinn et al. (111) showed that T_FH_ cells induced eLF formation in a manner that required the CXCL13-mediated homing of circulating memory T_FH_ cells (90). In line with these findings, the use of a blocking antibody to target CXCL13 could theoretically prevent eLF formation in humans. However, this species-specific functional difference calls the translational nature of the proposed axis into question.

Despite these limitations, clinical evidence still provides a strong argument for the involvement of eLFs in driving progression. Furthermore, the findings derived from EAE studies still absolutely merit consideration and could still provide critical insight into this potential link.

## If The Shoe Fits: Evidence Supporting a Role For eLFs in MS

The persistent interrogation of the composition of treated and untreated patient blood, serum, and CSF continues to reveal new biomarkers and implicate new B and T cell subsets and functions contributing to disease severity. These insights have consequently necessitated an evolving, flexible view of the mechanisms underlying MS pathogenesis and progression. Along these lines, a plethora of cytokines and cell types upregulated in MS patients strongly implicate eLFs as drivers of disease progression.

### T_**H**_17 Cells: Jack of All Trades

The functions and phenotypes of CD4^+^ T helper subsets have canonically been viewed simplistically, with each subset associated with a handful of signature cytokines, chemokine receptors, and typically a single transcription factor. However, CD4^+^ T cells are now known to display a remarkable degree of plasticity and versatility, qualities exemplified by T_H_17 cells.

T_H_17 cells are thought to be the primary T helper subset driving MS pathogenesis. Initially described in EAE models, this hypothesis is also supported in humans as T_H_17 are elevated in the CSF of MS patients, specifically during relapse (91). Several T_H_17-associated cytokines are associated with MS pathology. One study showed an increase in IL-22, a cytokine which coincidentally shares with IL-17 the ability to promote BBB breakdown, in the serum of patients experiencing relapse (112). Moreover, IL-6 and IL-23, both of which are required for T_H_17 maturation and maintenance, are also overrepresented in the CSF of MS patients (113, 114).

As detailed above, infiltration of the CNS is tightly regulated and varies depending on both the point of entry as well as on the inflammatory context. CCR6, a chemokine receptor that is required to cross the blood-CSF barrier in the choroid plexus, is highly expressed by T_H_17 cells (115, 116). Additionally, CCL20, the ligand of CCR6, was recently found to be upregulated in the CSF of MS patients (117). Taken together, the strong association of numerous cytokines and chemokines specifically related to the T_H_17 subset makes the CNS of MS patients an auspicious locale for the function and persistence encephalitogenic T_H_17 cells.

In addition to the more overt pathogenic contributions of this subset, several recent findings have suggested that T_H_17 cells might play a more inconspicuous role, namely in orchestrating GC-like responses and inducing the formation of meningeal eLFs in MS.

T_H_17 cells are known to secrete large amounts of IL-21, a cytokine typically secreted by T_FH_ cells (118, 119). T_FH_ cells, which are known to be upregulated in MS patients, are a specialized subset required for directing B cell responses within the GC reaction, such as proliferation, CSR, and differentiation into memory B cells and ASCs. Since IL-21 is primarily associated with T_FH_ cells, it would stand to reason that T_H_17 cells may have the capacity to function in a T_FH_-like capacity. Indeed, a study by Mitsdoerffer et al. (120) which showed that, upon adoptive transfer to T cell-deficient mice, T_H_17 cells were able to initiate GCs, promote isotype switching, and induce a pronounced antibody response, confirmed this theory. Further establishing the B-helper capacity of T_H_17 cells, a recent study showed that IL-17, when combined with BAFF, a cytokine also upregulated in MS patients, promoted B cell survival, proliferation, and differentiation into PCs, providing a second method by which T_H_17 cells can promote GC-like B cell responses within the CNS (120).

The ability to induce eLF formation has been shown in other contexts such as iBALT formation and occurred in an IL-17-dependent manner (97). Likewise, this capacity was also demonstrated in an EAE study that showed that adoptive transfer of MOG-reactive T_H_17 cells induced the formation of eLFs within the CNS through stimulating the production of CXCL13 by stromal cells (121). In a model of spontaneous arthritis, T_H_17-derived IL-17 was also shown to be critical for the development of autoreactive GCs (122). A separate study also showed the ability of IL-17 to induce meningeal fibroblast remodeling *in vivo* and *in vitro* (123). In mucosal tissues, IL-22 was also shown to induce eLF formation by stimulating the production of homeostatic chemokines by stromal cells (124, 125). This introduces the possibility that the elevated levels of CXCL13 in the CSF of MS patients may be due to T_H_17-derived IL-17 and IL-22.

### B Cells: Bad Memories

Several chemokines and cytokines that are upregulated in the CSF of MS patients are known to be important for facilitating B cell migration, activation, differentiation, and survival. The inflamed CNS of MS patients therefore seems to provide a microenvironment that is particularly conducive for facilitating B cell-mediated responses.

Memory B cells, specifically a subset known as IgD^−^CD27^−^ “double-negative” (DN) memory B cells, are abnormally overrepresented in the peripheral blood and CSF of MS patients. This subset of memory B cells has also been associated with SLE and RA and is associated with disease severity (83). The pathogenic relevance of memory B cells in MS has been substantially corroborated by studies exploring the immunological aspects of previously established non-B-cell-targeting DMTs. Indeed, depletion of memory B cells was associated with the efficacy of IFN-β, glatiramer acetate (GA), and dimethyl fumarate (DMF) (126). Furthermore, the clinical success of natalizumab has also been associated with its ability to reduce the frequency of memory B cells in the CNS.

As previously stated, memory B cells from MS patients secrete abnormally high quantities of IL-6, TNFα, and GM-CSF (67). This particular milieu promotes inflammation by increasing the permeability of the CNS vasculature, stimulating the production of IL-6 and IL-12 by myeloid cells, and the maintenance of pathogenic T_H_17 effector responses. Of note, B cell-derived IL-6 has also been shown in a model of SLE to be required for the formation of spontaneous eLFs (127). A recent study also demonstrated that B cells from the CSF of RRMS and progressive MS patients secrete large amounts of VEGF and LTα, respectively. Importantly, both of these growth factors promote the development of eLFs by stimulating lymphangiogenesis (128). Additionally, the enhanced production of neurotoxic factors by B cells from MS patients could offer further explanation for the strong association between meningeal inflammation and subpial cortical damage.

DN memory B cells have enhanced APC functions, indicated by elevated levels of MHCII along with the costimulatory molecules CD80, CD86, and CD40 (129). This was demonstrated by a study which found that B cells from MS patients were able to activate T cells in the presence of neuroantigens, unlike B cells from healthy controls. Memory B cells within the CNS of MS patients therefore have an enhanced capacity to serve as potent APCs to encephalitogenic memory CD4^+^ T cells and strongly contribute to the proinflammatory milieu within the CNS. The reactivation of encephalitogenic CD4^+^ T cells will result in the reciprocal reactivation of the presenting memory B cell. It is important to note that while reactivation of memory B cells is thought primarily to result in ASC generation, these cells are also fully able to undergo further affinity maturation in secondary GC-like reactions.

In addition to their proinflammatory functions within the CNS, memory B cells from MS patients also seem to have enhanced brain-infiltrating potential. In line with the observations from natalizumab treatment, B cells from MS patients express high levels of very late antigen-4 (VLA-4) (130). These cells also express ICAM-1 and activated leukocyte cell adhesion molecule (ALCAM), both of which facilitate migration across the BBB and BCSFB (131, 132). Interestingly, the absence of B cells within the parenchyma is supported by the enhanced expression of molecules that preferentially facilitate migration through meningeal vasculature.

In summary, memory B cells in MS patients display several phenotypic and functional traits that support not only an enhanced ability to migrate to, stimulate, and perpetuate inflammatory responses within the meningeal spaces, but also to promote the formation of eLFs.

### T_FH_ Cells: Hurting More Than Helping?

T_FH_ cells are broadly identified as CXCR5^+^PD-1^+^BCL6^+^ICOS^+^ and their effector function is primarily associated with the secretion of IL-21 (91). This subset is most strongly linked to GC B cell responses and has only recently been included as a disease-relevant subset in MS and other disease conditions (133, 134). T_FH_ cells have been associated with RA, SLE, and Sjögren's syndrome in a manner dependent on their ability to support GC B cell responses (135). T_FH_ and B cells provide critical signals to each other including signals important for development, effector function, and survival. Important T_FH_-derived signals include IL-21, which stimulates CSR, CD40L, which delivers costimulatory signals via CD40, and inducible T cell costimulator (ICOS). Secretion of BAFF, a potent B cell survival factor, by mouse T_FH_ cells was reported; however, BAFF secretion has so far not been reported by human T_FH_ cells (136). Additionally, a link between T_FH_ cells and AID expression in GC B cells has been suggested.

GWAS studies have shown that polymorphisms in IL-21, CXCR5, and PD-1 are genetic risk factors for MS. Moreover, about 20% of the CD4^+^ T cells in the CSF of MS patients express CXCR5, and active lesions have been shown to contain IL-21^+^ as well as CD40L^+^ CD4^+^ T cells (102, 137). Additionally, IL-21, BAFF, and CXCL13 are all abnormally elevated in MS patients (89, 138). However, these observations only supported the involvement of T_FH_ cells indirectly, due to the fact that many of these markers are also linked to other cell populations that are known to play a role. For example, IL-21 is known to be secreted by T_H_17 cells, a significant and well-known driver of MS (139).

The link between T_FH_ cells and MS was solidified in a 2013 study by Christensen et al. (140) reporting an increase in T_FH_ cells in RRMS and SPMS as well as a correlation with progression. Importantly, the same study showed that the T_FH_ cells were ICOS^+^ and correlated with the frequency of PBs. A subsequent study also reported the elevation of T_FH_ cells in the blood of MS patients as well as a positive correlation with disease severity (133). In further support of the importance of T_FH_ cells in MS, several studies showed that circulating T_FH_ cells are among the cells that are most prominently affected by DMTs, including fingolimod, and abatacept (141, 142). These studies indicate that a decrease in circulating T_FH_ cells is a prominent feature accompanying positive clinical responses.

In light of this cumulative body of evidence, it is plausible that T_FH_ cells in MS patients play a substantial role in eLF formation. It is particularly intriguing that T_FH_ cells share many features with T_H_17 cells, including the secretion of IL-21 and the ability to support GC responses. In fact, similar to studies showing the ability of T_H_17 cells to become T_FH_ -like T_H_17 cells, T_FH_ cells can become T_H_17-like T_FH_ cells, which express the transcription factor RORγt, the chemokine receptor CCR6, and secrete IL-17 as well as IL-21 (143). Notably, T_H_17-like T_FH_ cells display a formidable ability to induce antibody production (143). In MS patients, DMF treatment was shown to decrease the frequency of T_H_17-like T_FH_ cells and increase that of T_H_2-like T_FH_ cells, giving credence to the relevance of this unique subset (144). This reinforces evidence that T_H_17-like T_FH_ cells were increased in PPMS patients (140).

Importantly, the potential humoral dysregulation resulting from an overrepresentation of T_FH_ cells may be further amplified in MS patients due to a decrease in T_FR_ cells, the regulatory counterpart of T_FH_ cells, reported in a recent study (145). Similar to what has been observed regarding T_regs_, T_FR_ cells in MS patients also exhibit reduced suppressive capacity, indicated by abnormal IgG production in the blood, and CSF (146). Notably, a recent study reported that eLFs found in SPMS patients are devoid of T_FR_ cells, despite detection in the CSF. The lack of T_FR_ was also shown in the less-defined eLF aggregates from PPMS patients (147).

Taken together, these observations strongly support a potential contribution of T_FH_ cells to the progression of MS, namely via stimulating eLF formation, orchestrating GC-like responses in the CNS, and providing signals that support multiple inflammatory populations in the CNS. Of particular interest is the ability to adopt T_H_17-like effector functions, for this consequently amplifies the encephalitogenic potential of T_FH_ cells. The complex and multifaceted involvement of T_FH_ cells may represent the next paradigm shift in the journey toward understanding the pathogenesis and progression of MS.

## The (Un)Usual Suspects: Unique Inflammatory Cell Subsets and Their Potential Contribution to eLF Formation

Studies attempting to elucidate the mechanisms underlying MS have led to a single unanimous conclusion: it's complicated. MS pathogenesis is a complex process involving a vast array of cell types, all of which vary in their phenotype and function, and thus their pathogenic contribution, depending on the stage of disease. However, the continuous advances in multiparametric analyses have enabled us to more closely interrogate the characteristics of specific immune cell subsets associated with MS. This ability has revealed two subsets of particular relevance to progression, potentially via their contribution to eLF formation: T_H_1-like T_H_17 cells and T-bet^+^ memory B cells. In this section, we will describe the distinguishing features of these subsets and the evidence supporting their involvement in MS pathogenesis and progression, with a particular focus on their role in eLF formation.

### T_H_17.1 Cells: Potently Detrimental

Mechanisms of T cell plasticity are still enigmatic, and attempts to define novel T helper subsets, though regularly proposed, often lack sufficient evidence to merit their inclusion in the established lineup. However, numerous studies have not only confirmed the existence of T_H_1-like T_H_17 cells, but also established their functional relevance in both pathogenesis and progression of MS.

T helper subsets are conventionally characterized by the expression of a signature transcription factor, cytokine, and chemokine receptor. As mentioned above, T_H_17 and T_H_1 cells are considered the two primary encephalitogenic T helper subsets in MS and are identified as RORγt^+^IL-17^+^CCR6^+^ and T-bet^+^IFN-γ^+^CXCR3^+^, respectively.

T_H_1-like T_H_17 cells are a recently described subset of T_H_17 cells that, as the name suggests, express both T_H_1- and T_H_17-associated signature molecules and are identified as T-bet^+^RORγt^+^IFN-γ^+^IL-17^+^CXCR3^+^CCR6^+^. T_H_1-like T_H_17 cells have been identified in MS lesions and are selectively expanded in RRMS patients with more severe disease (148).

Recently, a variant of this subset, distinguished primarily by the additional expression of GM-CSF, was identified. Termed T_H_17.1 cells, this subset is associated specifically with early disease activity and correlated with the transition from clinically isolated syndrome (CIS) to clinically definite MS (CDMS). This observation is in line with the known role of GM-CSF as a critical proinflammatory mediator early in disease.

It is important to note that, although all three cytokines are expressed, T_H_17.1 cells express a relatively lower amount of IL-17. Therefore, the authors posit that IFN-γ and GM-CSF are considered the major proinflammatory cytokines responsible for association with the transition from CIS to CDMS. Interestingly, T_H_17.1 cells isolated from the CSF of relapsing patients express IL-17 to a degree similar to IFN-γ, while GM-CSF expression is decreased, suggesting that IL-17 is more important for progression than for onset. This shift in cytokine secretion may be attributable to the T_H_17-promoting milieu of the inflamed CNS increasing the production of IL-17, the regulation of which is antagonistic to that of GM-CSF in humans (149). Alternatively, this could be a result of IL-21 signaling, which induces the downregulation of T-bet and GM-CSF (149, 150).

In addition to the secretion of proinflammatory cytokines known to be elevated in MS patients, the encephalitogenicity of T_H_17.1 cells is also attributed to the enhanced brain-homing potential imparted by the simultaneous expression of CXCR3, CCR6, and VLA-4, all of which are important for trafficking to the inflamed CNS. In support of this, CXCL10, CCL20, and VCAM-1, the corresponding ligands, are highly upregulated in the CSF of MS patients during inflammation. Interestingly, T_H_17.1 cells have also been shown to acquire CCR2 expression as disease progresses. The expression of this additional receptor further amplifies this subset's ability to infiltrate the CNS. In further support of their clinical relevance, a recent study investigating the effects of DMF on immune cell populations found that T_H_17.1 cells are indeed downregulated as a result of treatment (144). Additionally, T_H_17.1 cells were shown to be markedly accumulated in the blood of patients who clinically responded to natalizumab treatment, further implicating their pathogenicity (151).

### ABC's and OCB's: T-Bet^+^ DN Memory B Cells and Their Potential Role in eLFs

The evidence that OCBs target ubiquitous intracellular self-antigens in a patient-specific manner would suggest that these likely originate in response to dead cell debris (49). Therefore, since OCBs are considered to be derived from the reactivation of DN memory B cells, this begs the question: where did these autoreactive memory B cells come from? Autoreactive B cells are present in the periphery of healthy individuals, but their aberrant activation and subsequent differentiation into memory B cells or autoantibody secreting PBs and PCs is prevented through several peripheral tolerance mechanisms within the GC. Therefore, the existence of memory B cells with such reactivity indicates a breach of these GC-related peripheral tolerance mechanisms (147).

As detailed above, DN memory B cells are considered a major pathogenic cell type in MS. However, similar to other immune cell types, more in-depth characterization of memory B cells has revealed several functionally distinct subsets, including recently described “atypical” memory B cell subsets. Atypical memory cells expressing CD11c and T-bet are associated with autoimmune diseases including SLE (152). Although initially described as age-associated B cells (ABCs), these cells are present in both healthy donors as well as aged mice and humans (153, 154). Functionally, these T-bet^+^ memory B cells are excellent APCs (155).

T-bet^+^ memory B cells are thought to be generated in a manner similar to extrafollicular responses (156). The expression of T-bet in B cells is induced by IFN-γ stimulation. The inflamed CNS of MS patients provides a microenvironment that supports the differentiation and persistence of these cells, due to the abundance of IFN-γ. Additionally, the reactivity of OCBs toward antigens derived from dead cell-debris provides evidence that the inflamed CNS also contains molecules that can stimulate Toll-like receptor 9 (TLR9), the second signal required for T-bet^+^ memory B cell development. Indeed, in all categories of MS, T-bet^+^ memory B cells, are elevated. Importantly, T-bet^+^ memory B cells display the same proinflammatory attributes that have been described for memory B cells in MS. In addition, T-bet^+^ memory B cells also express high amounts of CD20 and is therefore a major target of BCDTs. T-bet expression is also strongly associated with IgG1 and IgG3 class-switching, which are isotypes that are associated with MS (157).

The expression of CXCR3 in addition to CXCR5 enhances the brain-homing potential of these cells, enabling migration toward CXCL10 and CXCL13, both of which are elevated in the CSF of MS patients. Additionally, *in vitro* studies have demonstrated an enhanced ability of T-bet^+^ memory B cells to migrate through human brain endothelial layers. Similar to what was found regarding T_H_17.1 cells, T-bet^+^ memory B cells also accumulated in the blood of natalizumab-treated patients.

Although the enhanced antigen-presenting capabilities and proinflammatory characteristics of memory B cells from MS patients have been well-established, these new findings provide further evidence supporting the likelihood that T-bet^+^ memory B cells reactivate encephalitogenic CD4^+^ T cells in the brain. Importantly, a recent study showed that DN memory B cells from MS patients express ICOSL at levels only slightly lower that of mature naïve B cells (158). This enables direct interaction with T_FH_ cells within the CNS, which, in concert with the milieu of the inflamed CNS may promote meningeal eLF formation and propagate GC-like responses therein.

## Future Perspectives: Potential Novel Approaches to Targeting eLFs in MS

Despite the vast progress made regarding our understanding of MS, the ability to halt disease progression remains an elusive and enigmatic target. Several currently approved DMTs target B cells and may affect development of eLFs in MS; however, effects on eLFs may be limited by their access to the CNS. Nevertheless, the collaboration between T_FH_ cells and memory B cells, which underlies eLF formation, offers attractive therapeutic targets, especially in light of evidence implicating eLFs in driving MS progression. In this section, we will describe potential novel approaches to prevent formation of eLFs in MS by targeting B cells and T_FH_ cells.

### BTK Inhibitors

The rationale for pursuing therapeutics that selectively target B cells is clear, given the demonstrated efficacy of BCDTs. However, the inability of these mAbs to efficiently cross the BBB and BCSFB poses a significant problem in the treatment of the progressive forms of MS, as these barriers do not exhibit the same degree of permeability as seen in earlier disease stages. As a result, much interest has centered on pursuing compounds that can penetrate the intact BBB and BCSFB in the CNS of progressive MS patients, of which Bruton's tyrosine kinase (BTK) inhibitors have led the charge.

BTK is a tyrosine kinase that is essential for conveying signals necessary for B cell maturation, activation and survival, and BTK inhibitors showed efficacy in treating RRMS. Several different inhibitors are currently being investigated, including evobrutinib and PRN2246.

Evobrutinib has successfully completed phase 2 trials and showed positive results in treatment of RRMS (ClinicalTrials.gov number, NCT02975349) (159). Nevertheless, its degree of CNS penetration has not been assessed yet. However, PRN2246, another BTK inhibitor, can effectively penetrate the CNS and achieve therapeutic levels (160).

A third BTK inhibitor, fenebrutinib (GDC-0853), is very selective and potent as compared with previous inhibitors, and phase 3 clinical trials evaluating its efficacy in RRMS (FENhance 1 and FENhance 2) and PPMS (FENtrepid; ClinicalTrials.gov number, NCT04544449) are currently underway (161).

### Targeting T_FH_ Cells via CD28 and ICOS

The relevance of T_FH_ cells to MS has been established in both animal models as well as in human studies. As detailed above, their potential involvement in the progressive phase of MS via contributing to eLF formation makes them an attractive therapeutic target, specifically by exploiting the importance of the costimulatory receptors CD28 and ICOS for T_FH_ development and maintenance as well as for interacting with B cells (162, 163).

Abatacept is a fusion protein composed of the Fc region of human IgG1 and the extracellular domain of CTLA4, which binds CD80 and CD86, the ligands of CD28 as well as CTLA4. Abatacept has been efficacious in the treatment of RA, psoriasis vulgaris, and type 1 diabetes and is thought to act by abrogating autoimmune T cell responses through blocking costimulation through CD28. CD28 signaling is also thought to play a major role in T_FH_ cell development (164). Similar to MS, an increase in circulating T_FH_ cells has been associated with type 1 diabetes (165–167). A recent study showed that abatacept was able to decrease T_FH_ cells in a mouse model of type 1 diabetes, even after the disease is established (134). Furthermore, abatacept reduces circulating T_FH_ cells in RA and in Sjögren's syndrome (168, 169). While ACCLAIM, a phase 2 clinical trial studying the efficacy of abatacept in patients with RRMS, showed no clinical benefit, a subsequent study of samples obtained from patients participating in that trial showed that T_FH_ cells and T_reg_ cells were selectively decreased, the latter of which may be a significant disadvantage of this treatment (142, 170). Abatacept was followed by the development of belatacept, which has a higher affinity for both CD80 and CD86, and MEDI5256, which binds CD80 with greater affinity than CD86, although these have not been studied in the context of MS (171, 172). Interestingly, in a nonhuman primate model of transplantation, crosstalk between T_FH_ cells and B cells was more potently affected by treatment with a CD28 antagonist, FR104, compared to belatacept, suggesting that targeting CD28 directly might be more beneficial (162, 163, 172–175).

The increase in ICOS^+^ T_FH_ cells in MS is mirrored in several autoimmune diseases, such as SLE, Sjögren's syndrome, and type 1 diabetes (176–179). ICOS is a critical signal for T_FH_ cell development, functions such as IL-21 secretion, and is highly expressed on T_FH_ cells as well as on T_H_17 cells, albeit to a lesser extent (180, 181). Importantly, and in contrast to CD28 and CTLA4, ICOS expression is thought to be restricted to T_FH_ cells and antigen-experienced CD4^+^ memory T cells and is upregulated during reactivation (172, 182). In MS, the increase in IL-21- and ICOS-expressing CD4^+^ T cells would suggest a potential benefit in targeting ICOS-ICOSL interactions.

Prezalumab, a human mAb that binds ICOSL and blocks its interaction with ICOS, has been tested in SLE and arthritis; however results from a phase 2 clinical trial in patients with Sjögren's syndrome showed no clinical improvement and its development for the treatment of rheumatic diseases has been discontinued (162, 172). In light of this result and considering the restricted expression of ICOS to T_FH_ cells and CD4^+^ memory T cells, targeting ICOS might prove more effective than targeting ICOSL.

MEDI-570 is a mAb that binds ICOS, blocking its interaction with ICOSL. Additionally, MEDI-570 is afucosylated, a modification in the Fc region that enhances antibody-dependent cellular cytotoxicity by NK cells and macrophages (172, 183). In the context of autoimmunity, this mAb has only been evaluated in SLE; however the phase 1 study was terminated due to commercial considerations (ClinicalTrials.gov number, NCT01127321) (172). Nonetheless, the selective elimination of T_FH_ cells and CD4^+^ memory T cells, two CD4^+^ T cell populations strongly associated with disease activity, bolsters the rationale for further exploring this class of therapeutics.

Given the importance of both the CD28 and ICOS signaling pathways in T_FH_ cells, the recent development of a first-in-class dual inhibitor targeting CD28 and ICOS named ALPN-101 is particularly noteworthy, as it may offer the ability to compound the benefits observed using CD28 - and ICOS-targeting mAbs individually. While this compound has only completed phase 1 safety trials, preliminary evidence using an adoptive-transfer EAE model has yielded promising results as it was able to significantly ameliorate disease severity (ClinicalTrials.gov number, NCT03748836). An important consideration is that despite its molecular weight (80.8 kDa) being much smaller than that of traditional mAbs (~150 kDa), the BBB will likely still impede CNS access and limit its effectiveness in MS.

### Targeting eLF-Associated Molecules: IL-17, IL-22, IL-23, IL-21, and CXCL13

As described above, the induction of eLFs is coordinated by cytokines associated with T_FH_ cells and T_H_17 cells, all of which are overexpressed in MS. Both IL-17 and IL-22, which are produced by T_H_17 cells, facilitate BBB disruption and potentially induce the production of CXCL13 by meningeal stromal cells (111, 112, 125, 184). In EAE, these cytokines have also been shown to promote expansion of the reticular network (89, 121). A proof-of-concept study of secukinumab, a mAb targeting IL-17A, showed a reduction in annualized relapse rates in patients with RRMS (185). However, follow-up studies have not been reported. Currently, secukinumab and ixekizumab, a second anti-IL17A mAb, are approved for the treatment of psoriasis (186). A mAb targeting IL-22, fezakinumab, is available and has undergone clinical trials for psoriasis (ClinicalTrials.gov number, NCT00563524), RA (ClinicalTrials.gov number, NCT00883896), and atopic dermatitis (ClinicalTrials.gov number, NCT01941537); unlike secukinumab, this antibody has not been investigated in MS (187).

Meningeal stromal cells also secrete IL-23, required for T_H_17 maintenance, in inflammatory conditions (113, 114, 121, 188). IL-23 promotes the release of IL-22 by synovial fibroblasts in a model of arthritis (125). IL-23, which structurally shares the p40 subunit with IL-12, has been explored as a target for RRMS treatment in a phase 2 trial with ustekinumab, which targets p40 and thus exhibits dual specificity for IL-23 and IL-12 (189). Notably, guselkumab, a first-in-class mAb specific for the IL-23-exclusive p19 subunit and has been approved for treatment of psoriasis (190).

IL-21 is expressed by and induces the expansion of both T_FH_ cells and T_H_17 cells (191). IL-21 also promotes the generation of T-bet^+^ DN memory B cells (130, 156). An anti-IL-21 mAb, known as NNC01140006 or BOS161721, is currently being investigated in SLE in a phase 2 trial, but has not been explored in MS (ClinicalTrials.gov number, NCT03371251) (187, 192).

Lastly, CXCL13 is overexpressed in MS patients, strongly correlates with disease activity as well as the frequency of B cells and PBs in the CSF, and can be expressed by T_FH_ cells in humans. Reduction in CXCL13 levels in the CSF can be accomplished by several DMTs, including natalizumab and fingolimod (193, 194). Quinn et al. (91, 111) showed that blocking CXCL13 protected against disease development in the T_H_17-mediated adoptive transfer EAE model by reducing the influx of T_FH_ cells into the CNS, which resulted in a reduction of B cell-mediated inflammation in the CNS. Additionally, a neutralizing mAb directed against CXCL13, MAb 5261, inhibited CXCL13 function *in vitro* (195). However, it has not been explored in MS.

Given the association of these cytokines with MS and their ability to support the continuous recruitment and differentiation of inflammatory effector subsets, targeting these cytokines is an approach that warrants investigation. Importantly, efficient crossing of the BBB or BCSFB still remains a formidable hinderance to the efficacy of these drugs. Although therapeutic mAbs delivered intravenously can be detected in the CSF, the concentration is vastly smaller than that in the serum (196). RTX, for example, only reaches concentrations in the CSF < 0.1% of that in the serum (197). While intrathecal administration has been investigated, an abundance of efflux transporters such as the neonatal Fc receptor (FcRn) present on the BBB endothelium results in the rapid clearance of therapeutic monoclonal antibodies from the CSF into the blood, preventing the meaningful retention of these therapeutics within the CNS (198–201). Indeed, human and animal studies show that intrathecal RTX is rapidly cleared from the CSF and accompanied by a concomitant increase in serum concentration (197, 200, 202, 203). Thus, CNS penetration remains a paramount issue to address in the advancement of DMTs.

## Concluding Remarks

The insights afforded by the more in-depth characterization of disease-relevant immune and non-immune cell populations bring us closer to an understanding of the mechanisms driving MS relapses and progression. Specifically, the evidence supporting the interconnectedness of T_H_17, T_FH_, and B cells and the remarkable plasticity of each lineage could offer a possible inroad for unraveling the puzzle of the factors that induce and promote MS.

Ample evidence suggests that memory B cells in MS patients are ideally equipped for the reactivation of encephalitogenic CD4^+^ T cells, a process which can occur in the CNS or in the dCLNs. In the CNS, the inflammatory microenvironment that results from the reactivation of CD4^+^ T cells can stimulate the CXCL13-mediated recruitment of T_FH_ cells to the CNS, which is a particularly important link in the context of progression, due to the strong association of this subset with eLFs seen in other chronic inflammatory autoimmune diseases.

Importantly, CXCL13 will also result in the recruitment of naïve B cells into the CNS. As the inflammation in the CNS persists, it is possible that these infiltrating naïve B cells could encounter dead cell debris containing myelin-derived proteins and nucleic acids, the latter as potent ligands for TLR9 and TLR7. The combination of these signals along with those received from IFN-γ and T_FH_ cell-derived IL-21, will result in the T cell-independent generation of proinflammatory T-bet^+^ DN memory B cells (130). The generation of these autoreactive clones has major implications for subsequent relapses, as these cells are now not only more adept in their capacity to infiltrate the CNS but they are also potent APCs that can potentially precipitate a secondary break in CD4^+^ T cell tolerance. This can lead to the development of GC-like reactions and the expansion of further autoreactive B and T cell clones (204). These autoreactive responses are well-supported in the MS CNS due to the presence of proinflammatory cytokines and the abundance of BAFF, which is known to be elevated in MS (130). Furthermore, in the absence of TLR signaling, these cells will preferentially differentiate into PBs upon stimulation with IFN-γ and IL-21, thus representing a source of OCBs that may be unrelated to CNS autoantigens (204). This would be in line with a recent study that suggested that novel OCBs in RRMS patients result from the clonal expansion of memory and PB/PC populations in the CSF (84).

As stated previously, these cells express ICOSL at levels slightly lower than naïve B cells. The expression of ICOSL is noteworthy in light of a recent study which found that naïve B cells are able to reactivate effector memory CD4^+^ T cells from SLE and RA patients in an ICOSL-dependent manner even in the absence of T cell receptor triggering (205). Even more significant is that ICOSL preferentially stimulates effector T cells to produce IFN-γ, IL-17, and IL-22, all of which are highly expressed in the inflamed CNS of MS patients. While it must be noted that the effector memory cells expressed CD69, indicating they were recently activated, this finding nonetheless has important implications in the context of MS and could suggest that the reactivation of encephalitogenic CD4^+^ T cells can be carried out not only by non-cognate T-bet^+^ DN memory B cells, but also by naïve B cells, which are present in the inflamed and steady-state CNS. The result would be a population of autoreactive B and T cell clones that would expand with each relapse (206).

While this concept is speculative, the identification of these subsets and the evidence supporting their association with MS, specifically regarding reactivations that initiate relapses, provides a new lens through which we could view the inflammatory events that lead up to progression. Considering the paucity of inflammation during the progressive phase of MS, these potential mechanisms may be superseded by other mechanisms during those stages. Nonetheless, these findings help shed light on which cell populations may have an important impact on promoting relapses and would thus represent promising therapeutic targets.

## Author Contributions

AN, OS, and TF wrote and revised the manuscript. All authors reviewed and approved the manuscript.

## Conflict of Interest

The authors declare that the research was conducted in the absence of any commercial or financial relationships that could be construed as a potential conflict of interest.
